# On Asymptotic Efficiency of the *M*_2_*M*_4_ Signal-to-Noise Estimator for Deterministic Complex Sinusoids

**DOI:** 10.3390/s21154950

**Published:** 2021-07-21

**Authors:** Gianmarco Romano

**Affiliations:** Department of Engineering, University of Campania “L. Vanvitelli”, 81031 Aversa, CE, Italy; gianmarco.romano@unicampania.it

**Keywords:** signal-to-noise ratio (SNR) estimation, method of moments, asymptotic variance, estimator’s efficiency, super-efficiency, Cramer-Rao bound

## Abstract

The moment-based $M_{2}M_{4}$ signal-to-noise (SNR) estimator was proposed for a complex sinusoidal signal with a *deterministic* but unknown phase corrupted by additive Gaussian noise by Sekhar and Sreenivas. The authors studied its performances only through numerical examples and concluded that the proposed estimator is asymptotically efficient and exhibits finite sample super-efficiency for some combinations of signal and noise power. In this paper, we derive the analytical asymptotic performances of the proposed $M_{2}M_{4}$ SNR estimator, and we show that, contrary to what it has been concluded by Sekhar and Sreenivas, the proposed estimator is neither (asymptotically) efficient nor super-efficient. We also show that when dealing with deterministic signals, the covariance matrix needed to derive asymptotic performances must be explicitly derived as its known general form for random signals cannot be extended to deterministic signals. Numerical examples are provided whose results confirm the analytical findings.

## 1. Introduction

In [1], the authors address the problem of estimating the signal-to-noise ratio (SNR) in the case of a complex sinusoidal signal with a *deterministic* but unknown phase corrupted by additive Gaussian noise. A general signal model that, for example, represents the complex envelope of frequency modulated signals with deterministic modulating signals. The problem is found in many fields of practical interest such as in digital communication and radar systems (see, for example, [1] and references therein). The authors propose an estimator based on the sample-moment estimators of the second and fourth orders ($M_{2}M_{4}$ SNR estimator) and study its performances through numerical examples. The results let them conclude that the proposed SNR estimator is asymptotically efficient and presents finite sample super-efficiency in some cases that depend on the values of the signal and noise power.

In this paper, we show that the proposed SNR estimator is neither (asymptotically) efficient nor super-efficient, in any case. In fact, we study the performances of the estimator through asymptotic analytical analysis besides numerical examples and show that, though the estimator is not efficient, the performances are acceptable in many cases of interest and not too distant from the Cramer–Rao lower bounds (CRLB).

The $M_{2}M_{4}$ SNR estimator belongs to a wider class of moment-based SNR estimators that have been proposed mainly in the context of modulated telecommunication signals. As reminded in [2], a similar estimator was first proposed by Benedict and Soong in real AWGN channels [3]. Subsequently, in [4], Matzner derived a similar expression and, in [5], together with Englberger, obtained the same result using a different approach. In [6], the estimation of SNR for non-constant modulus constellations over a frequency-flat fading channel is considered, and a family of estimators that use higher-order moments is derived, and their performances studied. More specifically, the authors show that the $M_{1}M_{2}$ and $M_{2}M_{4}$ estimators contained in the family achieve the corresponding CRLBs for constant modulus constellation, while for multilevel constellations, performances degrade as the SNR increases. A different family of moment-based SNR estimators that makes use of the second, fourth and sixth moments is proposed in [7], with the purpose to improve the performances in the case of non-constant modulus constellations for increasing SNRs. Subsequently, in [8], the authors propose an extended family of moment-based SNR estimators that makes use of higher-order moments to further improve the performances with non-constant modulus constellations. In [1], a different signal model is considered, as the authors assume a complex exponential with a deterministic but unknown phase rather then randomly modulated signals. In practice, the authors consider a signal model very similar to the *M*-PSK vector model for the AWGN channel, with the only difference being the deterministic nature of the unknown phase. Under this assumption, the derivation in [1] is quite cumbersome. We show in this paper that a more general method can be used instead, as suggested by Kay [9]. The result is an $M_{2}M_{4}$ SNR estimator that has the same form of the similar estimator obtained for *M*-PSK constellations.

The $M_{2}M_{4}$ SNR estimator is simple to implement, has low computational complexity and is blind as it does not require prior knowledge of signal or noise. However, the performance of this estimator must be evaluated by numerical examples with Monte Carlo simulations and/or asymptotic analytical analyses. In fact, exact analysis is difficult as mathematics quickly becomes intractable as it involves the transformation of random variables to obtain the estimator pdf. In [6,7,8], the performances of the proposed moment-based estimators are studied in terms of asymptotic variance and CRLBs. However, care is needed when analytical asymptotic performances are derived in the deterministic case, since, in general, it is not possible to extend the results obtained for the random signals to deterministic signals, and in this paper, we extend to the complex case what has been already shown in the case of real deterministic a sinusoid in [10]. Since the derivation of asymptotic variances depends on a covariance matrix that, in turn, depends on the signal model, we show that, in general, the covariance matrix must be derived explicitly in the case of deterministic signals. Nevertheless, it turns out that, in the specific case of complex sinusoids with deterministic phase, the two covariance matrices coincide.

The paper is organized as follows: in Section 2, it is shown the derivation of even-order moments and estimators for signal and noise power as well as the SNR; in Section 3, asymptotic variances for all the estimators under investigation are derived; details of derivations are presented in Appendix A and Appendix B; we provide some numerical examples in Section 4; finally, in Section 5, we draw the conclusions.

## 2. Moment-Based Estimators

Let us consider the sequence of *K* samples

(1) $$y_{k}=\sqrt{S}x_{k}+w_{k},$$

where $x_{k}=e^{j\phi_{k}}$, and $\phi_{k}\in \left[0,2\pi \right)$ is a deterministic unknown sequence, *S* is a real positive scalar, and $w_{k}$ is an additive complex Gaussian random noise with zero mean and variance equal to *N*, i.e., $w_{k}∼\mathrm{CN}\left(0,N\right)$. The general model (1) includes, as special case, $\phi_{k}=2\pi \nu_{0}k$, i.e., a pure complex sinusoid with normalized frequency $\nu_{0}$; other choices for $\phi_{k}$ are possible as shown, for example, in [1]. The base assumption made here is that $\phi_{k}$ is deterministic in contrast, for example, to the case of phase modulated signals where the phase is random [2].

The signal-to-noise ratio (SNR), defined as ρ≜S/N, is unknown and represents the parameter we wish to estimate when the instantaneous phase $\phi_{k}$ is also unknown. In [1], an estimator for ρ based on the method of moments, built as a ratio of the estimators of *S* and *N* and that employs the second and fourth moment of $y_{k}$ ($M_{2}M_{4}$ estimator has been proposed, and its performances presented through numerical examples only. In this paper, we show that the same $M_{2}M_{4}$ estimator can be derived in an alternative, more general way, and we present asymptotic analytical performances, along with new numerical examples, that contradict some of the results obtained in [1].

The method of moments is a well-known general statistical method to derive estimators as a function of high-order sample-moments [9]. The key idea is to express the parameter to be estimated as a function of the moments of $y_{k}$ of different orders, i.e., $\mu_{m}=E\left[{\left|y_{k}\right|}^{m}\right]$, where *m* is an integer, and use the natural estimators of the moments ${\hat{\mu }}_{m}≜\left(1/K\right)\sum_{k=0}^{K-1}{\left|y_{k}\right|}^{m}$ in place of the true moments $\mu_{m}$ to obtain an estimate of the parameter from the observed samples. Such estimators typically present good statistical performances that can be derived through either numerical simulations or asymptotic analytical methods. In general, the asymptotic efficiency of an estimator derived using this method cannot be guaranteed; however, in many cases, the estimator turns out to be consistent.

The method relies on the possibility of expressing the moments $\mu_{m}$ as a function of the parameters to be estimated, i.e., *S* and *N* in this case. However, when in the observed signal model, one term is deterministic, as in the signal model (Equation (1)), the derivation of the moments might be cumbersome, as shown, for example, in [1]. For these cases, Kay [9] suggests to assume a random nature for the “deterministic” unknown phase. In this paper, we follow such an approach to obtain the same $M_{2}M_{4}$ estimator derived in [1] by assuming that $\phi_{k}$ is an r.v. independent of $w_{k}$. As consequence, the general closed form expression for the the even-order moments of $y_{k}$, $\mu_{2m}$, obtained in [7] for general QAM signals, applies also for the signal model (Equation (1)), i.e.,

(2) $$\begin{array}{cc} \mu_{2m}≜E[|y_{k}{|}^{2m}] & =\sum_{n=0}^{m}\frac{{\left(m!\right)}^{2}}{\left(m-n\right)!{\left(n!\right)}^{2}}E\left[|x_{k}{|}^{2n}\right]S^{n}N^{\left(m-n\right)}. \end{array}$$

Note that Equation (2), obtained under the random phase assumption, is equivalent to the expression

(3) $$\mu_{2m}=\sum_{n=0}^{m}\frac{{\left(m!\right)}^{2}}{\left(m-n\right)!{\left(n!\right)}^{2}}\left(\frac{1}{K}\sum_{k=0}^{K-1}{\left|x_{k}\right|}^{2n}\right)S^{n}N^{\left(m-n\right)},$$

under the assumption that K→∞. Since with $x_{k}=e^{j\phi_{k}}$, we have $E[|x_{k}{|}^{2n}]=1$ regardless of the distribution of $\phi_{k}$, Equation (2) simplifies to

(4) $$\mu_{2m}=\sum_{n=0}^{m}\frac{{\left(m!\right)}^{2}}{\left(m-n\right)!{\left(n!\right)}^{2}}S^{n}N^{(m-n).}$$

The above formula gives, as special cases, the second and fourth moments needed to form the $M_{2}M_{4}$ estimator,

(5) $$\begin{array}{ccc} \mu_{2} & = & S+N \end{array}$$

(6) $$\begin{array}{ccc} \mu_{4} & = & S^{2}+4SN+2N^{2}, \end{array}$$

from which we obtain both *S* and *N* as functions of $\mu_{2}$ and $\mu_{4}$ as follows

(7) $$\begin{array}{cc} S & =\sqrt{2\mu_{2}^{2}-\mu_{4}} \end{array}$$

(8) $$\begin{array}{cc} N & =\mu_{2}-S. \end{array}$$

Then, by using the sample moments

(9) $${\hat{\mu }}_{2}=\frac{1}{K}\sum_{k=0}^{K-1}{\left|y_{k}\right|}^{2}$$

(10) $${\hat{\mu }}_{4}=\frac{1}{K}\sum_{k=0}^{K-1}{\left|y_{k}\right|}^{4}$$

in place of the true moments in Equations (7) and (8), we obtain the following estimators for *S* and *N*

(11) $$\begin{array}{cc} \hat{S} & =\sqrt{\left|2{\hat{\mu }}_{2}^{2}-{\hat{\mu }}_{4}\right|} \end{array}$$

(12) $$\begin{array}{cc} \hat{N} & ={\hat{\mu }}_{2}-\hat{S}, \end{array}$$

where we apply the modulus to ensure that the estimates are always real and positive. Finally, the above estimators are then used to form the $M_{2}M_{4}$ SNR estimator

(13) $$\hat{\rho }=\frac{\hat{S}}{\hat{N}}.$$

Not surprisingly, the estimator takes on the same form as the $M_{2}M_{4}$ estimator proposed for phase modulated signals [2]. However, it is important to note that when it comes to deriving asymptotic performances, as we show in the following section, we cannot assume a random nature for the deterministic signals, and results obtained under such an assumption cannot in principle be readily extended to the signal model (1).

## 3. Asymptotic Performances

Analytical performances in terms of squared biases and variances of moment-based estimators can only be derived asymptotically for sufficiently large number of observed samples, i.e., K→∞. A method based on a first-order approximation analysis is suggested, for example, in [9], with the purpose of overcoming the mathematical intractability of the method that uses the transformation of random variables to obtain the pdf of the estimator. Alternatively, performances of the moment-based estimators may be obtained by estimating squared biases and variances through Monte Carlo simulations. We adopt both approaches, and we will show that, as expected, the analytical results confirm what we obtain through numerical simulations.

Let us start by defining the sample moment estimator over *K* observations as

(14) $$T_{m}\left(y\right)=\frac{1}{K}\sum_{k=0}^{K-1}{\left|y_{k}\right|}^{m}$$

and $g\left(T\right)$ is the function that maps the sample moments to the estimate, and T is the vector of the sample moments. More specifically, we define for the estimator $\hat{S}$, the function $g\left(T\right)=\sqrt{2T_{2}^{2}-T_{4}}$, with $T=\left(T_{2},T_{4}\right)$, as we use the second and fourth moments; the estimator $\hat{N}$ is defined by the function $h\left(T\right)=T_{2}-g\left(T\right)$; the estimator $\hat{\rho }$ is defined by the function $f\left(T\right)=g\left(T\right)/h\left(T\right)$.

In general, from the first-order Taylor expansion of g(T) about the point $T=E\left(T\right)=\mu$, we know that the mean of the estimator $g\left(T\right)$ is equal to $g\left(\mu \right)$, and by simple substitution using Equations (5) and (6), it can be shown that the estimators are asymptotically unbiased. The same applies to the other estimators defined by h(T) and $f\left(T\right)$, respectively.

We now turn our attention to variances. The first-order approximation of the variance of the estimator $\hat{S}$ is given by

(15) $$\mathrm{var}\left(\hat{S}\right)≃{\left.\frac{\partial g}{\partial T}\right|}_{T=\mu }^{T}C_{T}{\left.\frac{\partial g}{\partial T}\right|}_{T=\mu },$$

where $C_{T}$ is the covariance matrix of T that by definition is equal to

(16) $$C_{T}=E\left[\left(T-\mu \right){\left(T-\mu \right)}^{T}\right].$$

In general, for random signals $x_{k}$ in Equation (1), it is already known that each element of the covariance matrix is given by

(17) $${\left[C_{T}\right]}_{ij}=\frac{1}{K}\left(\mu_{2(i+j)}-\mu_{2i}\mu_{2j}\right),$$

where $\mu_{2m}$ is the true even-order moment. However, in the case of generic deterministic signals $x_{k}$, Equation (17) cannot be readily applied. Nevertheless, for the specific signal under consideration, i.e., the complex exponential signal with a deterministic unknown phase, it turns out that the elements of the covariance matrix can still be expressed by Equation (17), which is valid for any *K*, even for a small *K*. Details of derivation are shown in Appendix A.

In Equation (15), we also need the partial derivatives of $g\left(T\right)$, i.e.,

(18) $$\begin{array}{cc} \frac{\partial g}{\partial T_{2}} & =\frac{2T_{2}}{g\left(T_{2},T_{4}\right)} \end{array}$$

and

(19) $$\begin{array}{cc} \frac{\partial g}{\partial T_{4}} & =-\frac{1}{2}\frac{1}{g\left(T_{2},T_{4}\right)}. \end{array}$$

Similarly, we know that the first-order approximation of the variance of the estimator $\hat{N}$ is given by

(20) $$\mathrm{var}\left(\hat{N}\right)≃{\left.\frac{\partial h}{\partial T}\right|}_{T=\mu }^{T}C_{T}{\left.\frac{\partial h}{\partial T}\right|}_{T=\mu }$$

where

(21) $$\begin{array}{cc} \frac{\partial h}{\partial T_{2}} & =1-\frac{\partial g}{\partial T_{2}} \end{array}$$

(22) $$\begin{array}{cc} \frac{\partial h}{\partial T_{4}} & =-\frac{\partial g}{\partial T_{4}}. \end{array}$$

Finally, we have that the variance of the estimator $\hat{\rho }$ is well approximated by

(23) $$\mathrm{var}\left(\hat{\rho }\right)≃{\left.\frac{\partial f}{\partial T}\right|}_{T=\mu }^{T}C_{T}{\left.\frac{\partial f}{\partial T}\right|}_{T=\mu },$$

where

(24) $$\begin{array}{cc} \frac{\partial f}{\partial T_{2}} & =\frac{\partial g}{\partial T_{2}}\frac{1}{h}-g\frac{\partial h}{\partial T_{2}}\frac{1}{h^{2}} \end{array}$$

(25) $$\begin{array}{cc} \frac{\partial f}{\partial T_{4}} & =\frac{\partial g}{\partial T_{4}}\frac{1}{h}-g\frac{\partial h}{\partial T_{4}}\frac{1}{h^{2}}. \end{array}$$

Based on the same approach, it is possible to derive asymptotic performances for the sample moment estimators of order *m*. Let $g_{2m}\left(T_{2m}\right)=T_{2m}$, the function that maps the sample moments to the sample moment estimate, and from Equation (15), we have

(26) $$\mathrm{var}\left({\hat{\mu }}_{2m}\right)≃{\left.\frac{\partial g_{2m}}{\partial T_{2m}}\right|}_{T_{2m}=\mu_{2m}}^{T}C_{T_{2m}}{\left.\frac{\partial g_{2m}}{\partial T_{2m}}\right|}_{T_{2m}=\mu_{2m}},$$

where

(27) $${\left.\frac{\partial g_{2m}}{\partial T_{2m}}\right|}_{T_{2m}=\mu_{2m}}={\left.\frac{\partial }{\partial T_{2m}}\left(T_{2m}\right)\right|}_{T_{2m}=\mu_{2m}}=1$$

and the covariance matrix is

(28) $$C_{T_{2m}}=\frac{1}{K}\left(\mu_{4m}-\mu_{2m}^{2}\right).$$

Putting it all together

(29) $$\mathrm{var}\left({\hat{\mu }}_{2m}\right)≃\frac{1}{K}\left(\mu_{4m}-\mu_{2m}^{2}\right).$$

For convenience, we conclude this section by providing the Cramer–Rao lower bounds (CRLB) for all the estimators under analysis because they provide a significant benchmark for the performances of all corresponding estimators.

CRLBs for $\hat{S}$ and $\hat{N}$ are immediately given by the Fisher information matrix as derived in [1]. Note that in the derivation of the CRLB, the phases $\phi_{k}$ should be regarded as nuisance parameters. Thus, the true Fisher Information Matrix (FIM) has size (K+2)×(K+2). However, since $\phi_{k}$’s are uncoupled with *S* and *N*, the FIM turns out to be diagonal, and the final result does not change. The CRLBs are as follows:

(30) $$I\left(S,N\right)=\left(\begin{array}{cc} \frac{K}{2SN} & 0\\ 0 & \frac{K}{N^{2}} \end{array}\right)$$

and we have

(31) $$\begin{array}{cc} \mathrm{var}(\hat{S}) & \geq \frac{2SN}{K} \end{array}$$

(32) $$\begin{array}{cc} \mathrm{var}(\hat{N}) & \geq \frac{N^{2}}{K}. \end{array}$$

For $\hat{\rho }$ expressed in dB, i.e., ${\hat{\rho }}_{dB}$, we have

(33) $$\mathrm{var}\left({\hat{\rho }}_{dB}\right)\geq \frac{1}{K}{\left(\frac{10}{\ln10}\right)}^{2}\left(2\frac{N}{S}+1\right)\;{\mathrm{dB}}^{2}.$$

CRLBs for ${\hat{\mu }}_{2m}$, with m=1,2,…, are derived in Appendix B and can be written as

(34) $$\mathrm{var}\left({\hat{\mu }}_{2m}\right)\geq {\left(\frac{\partial \mu_{2m}}{\partial S}\right)}^{2}\frac{2SN}{K}+{\left(\frac{\partial \mu_{2m}}{\partial N}\right)}^{2}\frac{N^{2}}{K},$$

where

(35) $$\begin{array}{cc} \frac{\partial }{\partial S}\mu_{2m}\left(S,N\right) & =\sum_{n=0}^{m}\frac{{\left(m!\right)}^{2}n}{\left(m-n\right)!{\left(n!\right)}^{2}}S^{n-1}N^{\left(m-n\right)} \end{array}$$

and

(36) $$\begin{array}{cc} \frac{\partial }{\partial N}\mu_{2m}\left(S,N\right) & =\sum_{n=0}^{m}\frac{{\left(m!\right)}^{2}\left(m-n\right)}{\left(m-n\right)!{\left(n!\right)}^{2}}S^{n}N^{\left(m-n-1\right)}. \end{array}$$

## 4. Numerical Examples

In this section, we present some examples that show the performances of the proposed moment-based estimators for *S* and *N* and signal-to-noise ratio ρ in terms of squared bias and variance. We report estimated squared biases and variances obtained through Monte Carlo simulations, and we show that numerical results confirm the asymptotic performances derived in Section 3. We also compare them to the corresponding CRLBs, and we make some comments on the property of efficiency of the estimators.

For all estimators under study, we have estimated the squared bias and variance by averaging over $L=2^{13}=8192$ estimates on a varying number of observations *K*. The mean of each estimator is estimated by means of a standard sample mean estimator, e.g., for $\hat{S}$

(37) $$E\left[\hat{S}\right]\approx {\hat{\mu }}_{S}=\frac{1}{L}\sum_{l=1}^{L}{\hat{S}}_{l},$$

where ${\hat{S}}_{l}$ is a single estimate of *S* with *K* observations. The squared bias is then obtained by using the estimated mean in place of the true mean, e.g., $b^{2}\left(\hat{S}\right)={\left[E[\hat{S}]-S\right]}^{2}$. The variance of estimators is estimated by means of the standard sample variance estimator, e.g., for $\hat{S}$.

(38) $$\mathrm{var}\left(\hat{S}\right)\approx {\hat{\sigma }}_{S}^{2}=\frac{1}{L-1}\sum_{l=1}^{L}{\left({\hat{S}}_{l}-{\hat{\mu }}_{S}\right)}^{2}.$$

The estimation of squared bias and variance of estimators has been performed at different SNRs that, without loss of generality, are obtained by varying the power of the complex sinusoid *S* while maintaining the noise power fixed at N=4. The instantaneous phase of the complex exponential has been chosen to be $\phi_{k}=2\pi \nu_{0}k$, where *k* is the discrete-time index and $\nu_{0}=0.1234$. Note that the results do not depend on the specific form of the instantaneous phase, and other choices are possible, such as the phase used in [1].

In the following, we report results not only for the SNR estimator $\hat{\rho }$ but also for estimators of the second and fourth moments and for estimator of the signal power $\hat{S}$ and the noise power $\hat{N}$.

### 4.1. Performances of ${\hat{\mu }}_{2}$

**Figure 1:**
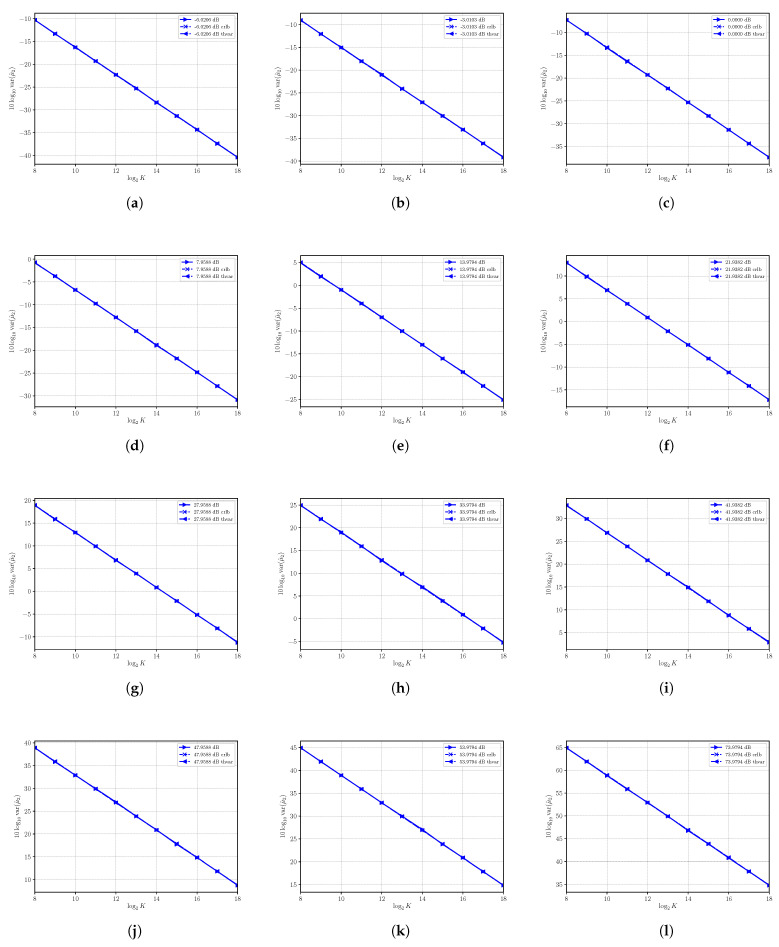
Asymptotic and estimated variances of the moment estimator ${\hat{\mu }}_{2}$ versus the number of observations *K*. Each sub figure (**a**–**l**) shows results for a given nominal value of SNR obtained by changing the signal power *S* and with noise power N=4.

The sample moment estimator ${\hat{\mu }}_{2}$ is, under first order approximation, unbiased. On the other hand, our focus is on the variance of ${\hat{\mu }}_{2}$, which has been estimated for several SNRs obtained by varying *S* with N=4. We report the results in Figure 1, where the corresponding CRLB and asymptotic performances are shown as well. For all the SNRs under consideration, we see that we have complete superposition of the three curves even for a small number of observations *K* and that the sample moment estimator is, then, always consistent and efficient. Though performances are always optimal, the variance tends to increase as the SNR increases.

### 4.2. Performances of ${\hat{\mu }}_{4}$

**Figure 2:**
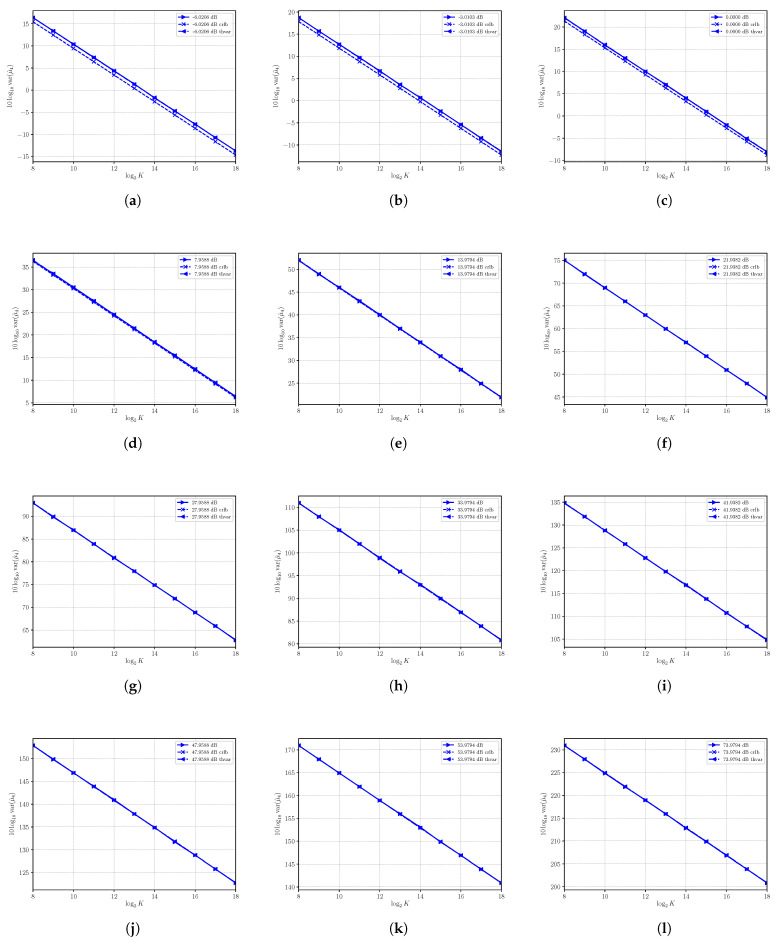
Asymptotic and estimated variances of the moment estimator ${\hat{\mu }}_{4}$ versus the number of observations *K*. Each sub figure (**a**–**l**) shows results for a given nominal value of SNR obtained by changing the signal power *S* and with noise power N=4.

The sample moment estimator ${\hat{\mu }}_{4}$ is, under first order approximation, unbiased. The performances in terms of variance of ${\hat{\mu }}_{4}$ are shown in Figure 2, where estimated variance, CRLB and asymptotic variance are shown at different SNRs obtained by varying *S* with N=4. In all cases under consideration, the estimated variance and the asymptotic variance are superimposed even for a small numbers of observation *K*, confirming, also in this case, the validity and usefulness of the asymptotic analysis carried out above. While in all cases the sample estimator ${\hat{\mu }}_{4}$ results are consistent, we see that at low SNRs, the estimator can be regarded as nearly efficient because the variance of ${\hat{\mu }}_{4}$ is not superimposed on the CRLB, though it is very close (as shown, for example, in Figure 2a–f). Note that the variance of the estimator increases to significantly large values as the SNR becomes larger by increasing the power *S*.

### 4.3. Performances of $\hat{S}$

**Figure 3:**
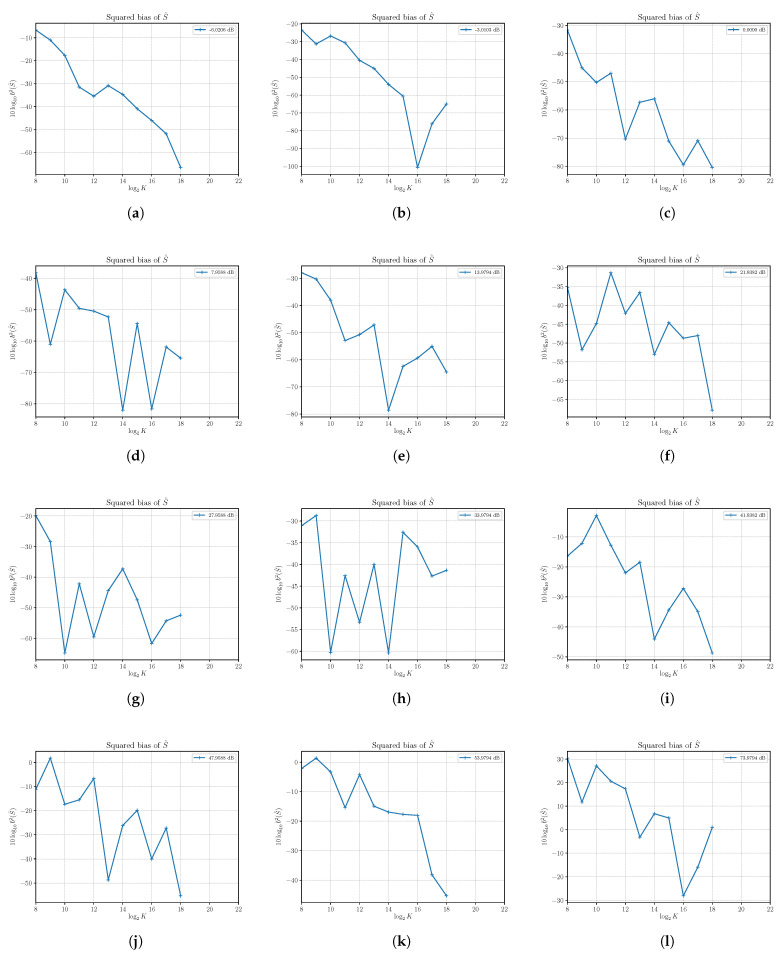
The estimated squared bias $b^{2}(\hat{S)}$ of signal power estimator $\hat{S}$ versus the number of observations *K*. Each sub figure (**a**–**l**) shows results for a given nominal value of SNR obtained by changing the signal power *S* and with noise power N=4.

In Figure 3, we report the results of the estimation of the squared bias of $\hat{S}$ at different SNRs obtained by setting N=4 and varying *S*. We see that in all reported cases, the squared bias is sufficiently small even for a small number of observed samples *K*, and then we find that $\hat{S}$ is practically (asymptotically) unbiased.

**Figure 4:**
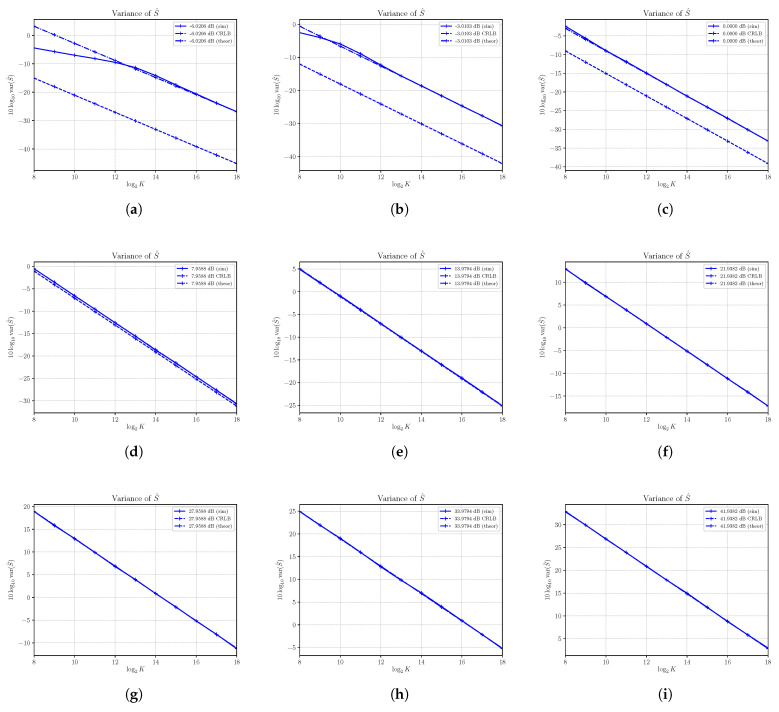

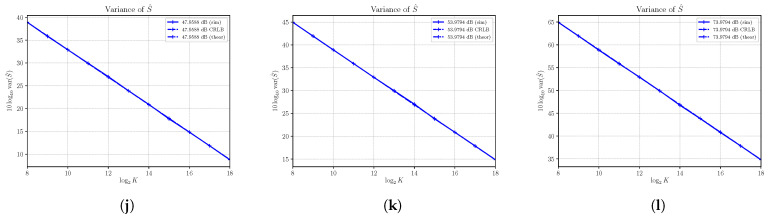
Asymptotic and estimated variances of the signal power estimator $\hat{S}$ versus the number of observations *K*. Each sub figure (**a**–**l**) shows results for a given nominal value of SNR obtained by changing the signal power *S* and with noise power N=4.

Estimates of the variance of $\hat{S}$ are shown in Figure 4 together with the corresponding asymptotic variances and CRLBs. First, the results confirm the derivation of the asymptotic performances obtained in Section 3. Though valid only for K→∞, it is interesting to note that the asymptotic results are achieved also for a small number of samples in most of the cases under consideration. In all cases, with $K=2^{18}$, the variance of $\hat{S}$ is well approximated by the asymptotic variance that shows that the estimator is consistent. It is not always efficient, especially at a relatively small SNR. Consider, for example, Figure 4a, which shows the variance for S=1 and N=4, corresponding to the SNR equal to −6.0206 dB. The asymptotic variance, which is achieved by the estimator with at least $K=2^{12}$ samples, cannot achieve the corresponding CRLB. This is also true in Figure 4b,c. On the other hand, starting from Figure 4d, we see that the asymptotic variance is superimposed to the corresponding CRLB, and then the estimator becomes efficient. For a sufficiently large SNR, the asymptotic variance is able to predict the effective variance of the estimator $\hat{S}$ even for a very small number of samples *K*.

### 4.4. Performances of $\hat{N}$

**Figure 5:**
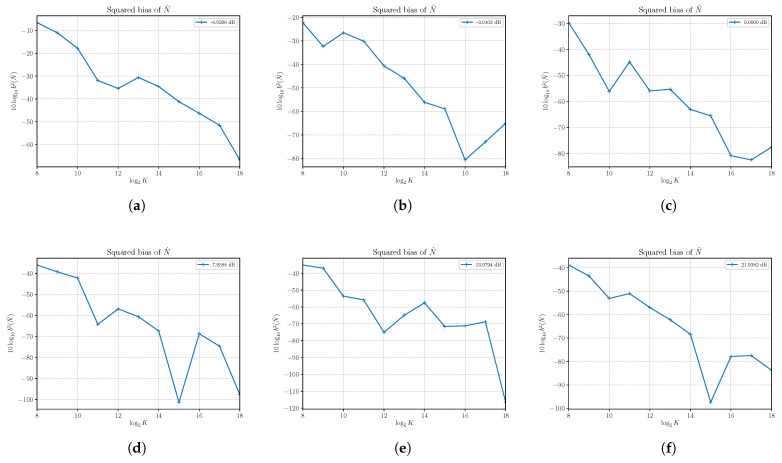

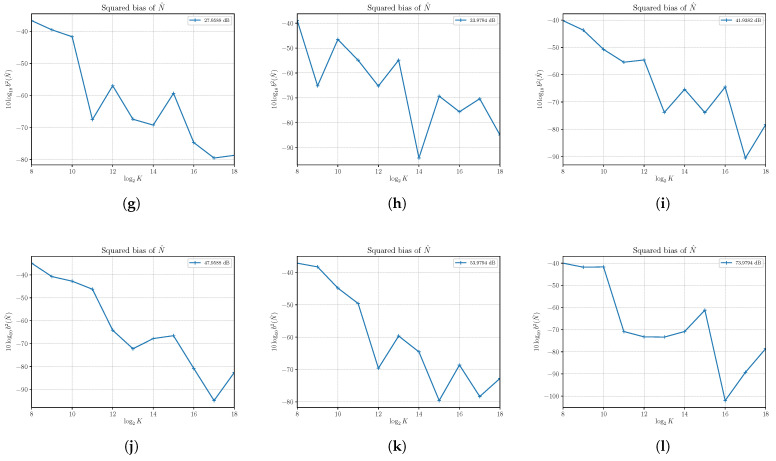
The estimated squared bias $b^{2}(\hat{N)}$ of the noise power estimator $\hat{N}$ versus the number of observations *K*. Each sub figure (**a**–**l**) shows results for a given nominal value of SNR obtained by changing the signal power *S* and with noise power N=4.

In Figure 5, the estimated squared bias of the estimator $\hat{N}$ at different SNRs with varying *S* and N=4 is shown. As for $\hat{S}$, we find that, in practice, we can consider $\hat{N}$ as an unbiased estimator, as expected by the first-order approximation analysis.

**Figure 6:**
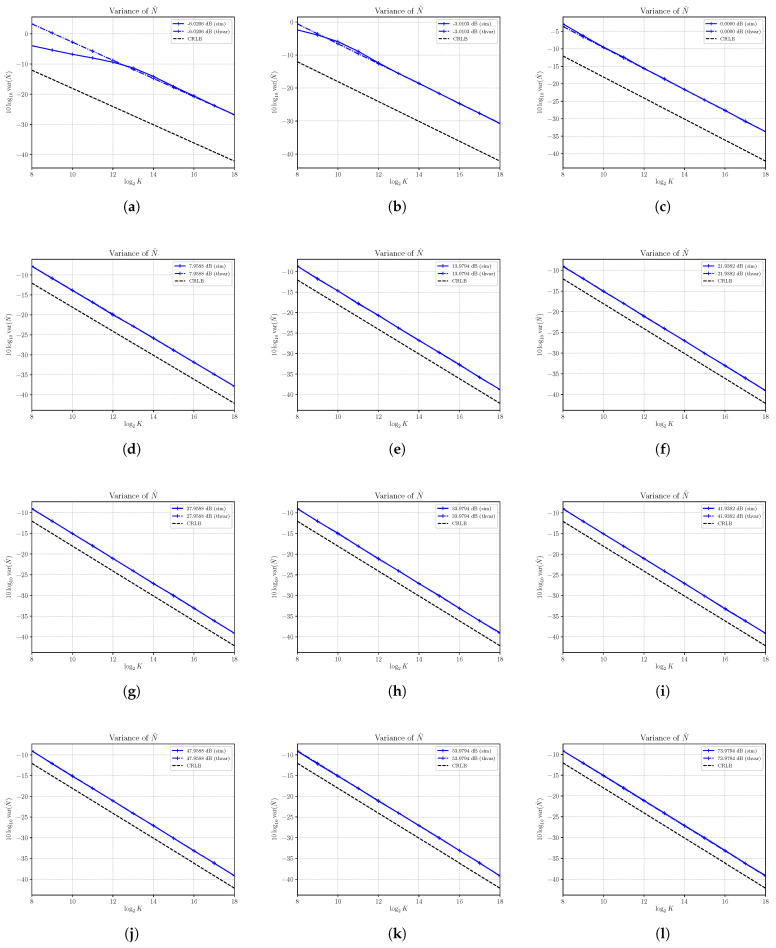
Asymptotic and estimated variances of the noise power estimator $\hat{N}$ versus the number of observations *K*. Each sub figure (**a**–**l**) shows results for a given nominal value of SNR obtained by changing the signal power *S* and with noise power N=4.

Under the same setup, we have obtained the estimation of the variance of $\hat{N}$, and we show the results in Figure 6 together with the asymptotic variances and the corresponding CRLBs obtained in Section 3. Note that we maintain the noise power fixed while varying the signal power *S*, and therefore, we have the same CRLB on all the figures.

In all cases, the estimated variances achieve the corresponding asymptotic variances, confirming the analytical results obtained in Section 3 and showing that the estimator is consistent.

In general, the asymptotic variance describes the current variance of $\hat{N}$ very well, even for a small number of samples, with the exception of some cases at low SNRs, as shown in Figure 6a–c. Furthermore, the variance never achieves the corresponding CRLB, and then the estimator is not efficient.

### 4.5. Performances of $\hat{\rho }$

**Figure 7:**
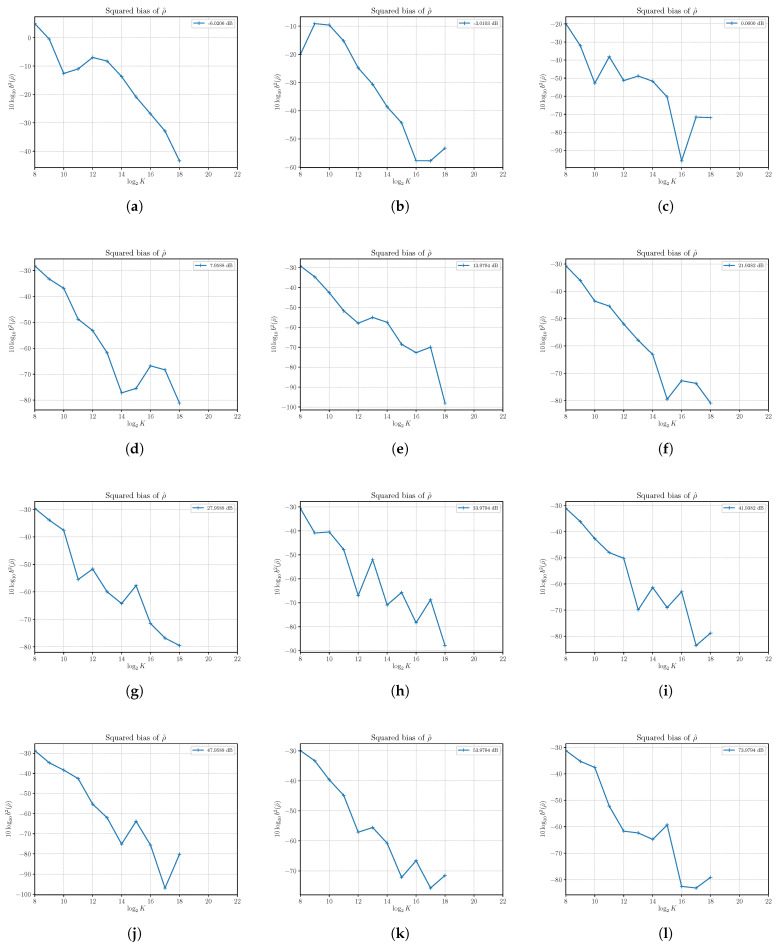
The estimated squared bias $b^{2}(\hat{\rho )}$ of the SNR estimator $\hat{\rho }$ versus the number of observations *K*. Each sub figure (**a**–**l**) shows results for a given nominal value of SNR obtained by changing the signal power *S* and with noise power N=4.

**Figure 8:**
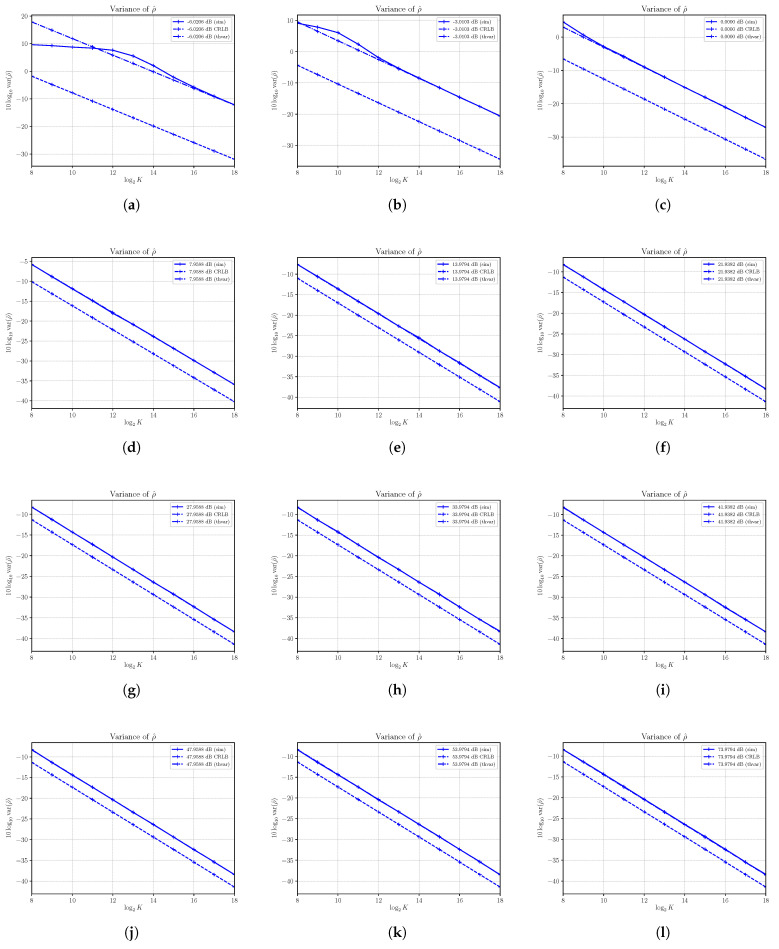
Asymptotic and estimated variances of the SNR estimator $\hat{\rho }$ versus the number of observations *K*. Each sub figure (**a**–**l**) shows results for a given nominal value of SNR obtained by changing the signal power *S* and with noise power N=4.

The estimated squared biases and variances of the SNR estimator $\hat{\rho }$ have been obtained for a number of different SNRs with Monte Carlo simulations run with N=4 and varying the signal power *S*, as conducted previously with $\hat{S}$ and $\hat{N}$. The results are reported in Figure 7 and Figure 8.

From the plots in Figure 7, we have confirmation that the estimator is practically unbiased.

In Figure 8, we report the estimated variances along with the corresponding asymptotic variances and CRLBs as derived in Section 3. First, the results confirm once again the validity of the analysis carried out in Section 3. In all cases, the estimated variances achieve the corresponding asymptotic variances, showing that the estimator is consistent. Note that the asymptotic variance is achieved with a small number of samples, with the exception of small SNRs, as we have already seen for the estimator of noise power $\hat{N}$. Clearly, the limits of the noise power estimator are also reflected in the performances of the SNR estimator as the SNR estimator is obtained as the ratio of $\hat{S}$ and $\hat{N}$. For the same reason, the estimator $\hat{\rho }$ cannot achieve the performances given by the CRLB, and then it is not efficient, though the difference between the variance of $\hat{\rho }$ and the corresponding CRLB is very small, especially at high SNRs.

The results obtained in this paper do not confirm what had been concluded in [1], where the authors stated that the same estimator was not only efficient, but in some cases, it was also super-efficient, i.e., with the variance of the estimator below the CRLB. The authors in [1] admit that the conclusion is based only on numerical results obtained in the case of S=100 and N=4 that corresponds to ρ=13.9794 dB and reported in Figure 4 in [1]. Specifically, in Figure 8e, it is shown that we have obtained in this paper the variance of $\hat{\rho }$ both numerically and mathematically with the same parameters used in Figure 4 in [1]. We have proven that the asymptotic value, which represents with very good approximation the variance of $\hat{\rho }$ even for a small number of samples, is always above the CRLB, and therefore, even in this specific case, the estimator is neither efficient or super-efficient.

## 5. Conclusions

The $M_{2}M_{4}$ SNR estimator for unknown deterministic complex phase signals proposed in [1] represents a useful tool for blind estimation of SNR. However, its performances, while acceptable in many applications of interest, are not those stated in [1]. In fact, through detailed analytical and numerical analysis of the performances presented in this paper, we could not confirm the finite sample super-efficiency or (asymptotically) efficiency of the $M_{2}M_{4}$ SNR estimator claimed in [1], which was based on numerical examples only.

In this paper, we have also derived analytical asymptotic performances for all the intermediate estimators that are used to form the $M_{2}M_{4}$ SNR estimator, namely even-order sample moments, signal and noise power. The results show that though the second and fourth sample moment estimators are efficient, as well as the estimator of the signal power for sufficiently high SNRs, the noise power estimator is not efficient. Consequently, the SNR is not efficient though its performances are close to the corresponding CRLB. In contrast to the case of signal and noise power, the performances of the SNR estimator do not degrade as the true SNR increases.

The derivation of analytical asymptotic performances revealed that, in general, it is not possible to readily extend the results obtained for random signals to the case of deterministic signals. More specifically, the covariance matrix required to compute the asymptotic variances needs to be explicitly derived. Nevertheless, in the specific case considered in this paper, i.e., complex sinusoid with deterministic phase, it turns out that the covariance matrix has the same form as that in the random case.

## Appendix A. Derivation of Covariance Matrix

In this section, we provide details of the derivation of Equation (17). Let us start from the definition of the covariance matrix, where each element is given by

(A1) $$\begin{array}{cc} {\left[C_{T}\right]}_{ij} & =\mathrm{cov}\left(T_{2i,}T_{2j}\right) \end{array}$$

(A2) $$\begin{array}{cc} \; & ≜E\left[\frac{1}{K}\sum_{k=0}^{K-1}|y_{k}{|}^{2i}\frac{1}{K}\sum_{p=0}^{K-1}{\left|y_{p}\right|}^{2j}\right]-E\left[\frac{1}{K}\sum_{k=0}^{K-1}{\left|y_{k}\right|}^{2i}\right]E\left[\frac{1}{K}\sum_{p=0}^{K-1}{\left|y_{p}\right|}^{2j}\right]. \end{array}$$

Now, consider the first term of the right-hand side. We get

(A3) $$\begin{array}{cc} E\left[\frac{1}{K}\sum_{k=0}^{K-1}|y_{k}{|}^{2i}\frac{1}{K}\sum_{p=0}^{K-1}{\left|y_{p}\right|}^{2j}\right] & =\frac{1}{K^{2}}\sum_{k=0}^{K-1}\sum_{p=0}^{K-1}E\left[{\left|y_{k}\right|}^{2i}{\left|y_{p}\right|}^{2j}\right] \end{array}$$

(A4) $$\begin{array}{cc} \; & =\frac{1}{K^{2}}\sum_{k=0}^{K-1}E\left[{\left|y_{k}\right|}^{2\left(i+j\right)}\right]+\frac{1}{K^{2}}\sum_{k=0}^{K-1}\sum_{p=0,p\neq k}^{K-1}E\left[{\left|y_{k}\right|}^{2i}{\left|y_{p}\right|}^{2j}\right] \end{array}$$

(A5) $$\begin{array}{cc} \; & =\frac{1}{K^{2}}\sum_{k=0}^{K-1}E\left[{\left|y_{k}\right|}^{2\left(i+j\right)}\right]+\frac{1}{K^{2}}\sum_{k=0}^{K-1}\sum_{p=0,p\neq k}^{K-1}E\left[{\left|y_{k}\right|}^{2i}\right]E\left[{\left|y_{p}\right|}^{2j}\right] \end{array}$$

Similarly, we obtain that the second term on the right-hand side of (A2) is

(A6) $$\begin{array}{c} E\left[\frac{1}{K}\sum_{k=0}^{K-1}{\left|y_{k}\right|}^{2i}\right]E\left[\frac{1}{K}\sum_{p=0}^{K-1}{\left|y_{p}\right|}^{2j}\right]=\\ \;\;\;\;\;\;\;\frac{1}{K^{2}}\sum_{k=0}^{K-1}E\left[|y_{k}{|}^{2i}\right]E\left[|y_{k}{|}^{2j}\right]+\frac{1}{K^{2}}\sum_{k=0}^{K-1}\sum_{p=0,p\neq k}^{K-1}E\left[|y_{k}{|}^{2i}\right]E\left[|y_{p}{|}^{2j}\right]. \end{array}$$

Putting it all together, we have that each element of the covariance matrix is

(A7) $${\left[C_{T}\right]}_{ij}=\frac{1}{K^{2}}\sum_{k=0}^{K-1}E\left[{\left|y_{k}\right|}^{2\left(i+j\right)}\right]-\frac{1}{K^{2}}\sum_{k=0}^{K-1}E\left[|y_{k}{|}^{2i}\right]E\left[|y_{k}{|}^{2j}\right].$$

The first term can be written explicitly as

(A8) $$\frac{1}{K}E\left[\frac{1}{K}\sum_{k=0}^{K-1}{\left|y_{k}\right|}^{2\left(i+j\right)}\right]=\sum_{p=0}^{i+j}\frac{{\left(\left(i+j\right)!\right)}^{2}}{\left(i+j-p\right)!{\left(p!\right)}^{2}}\left(\frac{1}{K}\sum_{k=0}^{K-1}{\left|x_{k}\right|}^{2p}\right)S^{p}N^{\left(i+j-p\right)},$$

while the second term becomes

(A9) $$\begin{array}{l} \frac{1}{K^{2}}\sum_{k=0}^{K-1}E\left[|y_{k}{|}^{2i}\right]E\left[|y_{k}{|}^{2j}\right]=\\ \;\;\;\;=\frac{1}{K}\sum_{k=0}^{K-1}\sum_{p=0}^{i}\frac{{\left(i!\right)}^{2}}{\left(i-p\right)!{\left(p!\right)}^{2}}|x_{k}{|}^{2p}S^{p}N^{\left(i-p\right)}\sum_{q=0}^{j}\frac{{\left(j!\right)}^{2}}{\left(j-q\right)!{\left(q!\right)}^{2}}{\left|x_{k}\right|}^{2q}S^{q}N^{\left(j-q\right)}=\\ \;\;\;\;=\frac{1}{K}\sum_{k=0}^{K-1}\sum_{p=0}^{i}\sum_{q=0}^{j}\frac{{\left(i!\right)}^{2}}{\left(i-p\right)!{\left(p!\right)}^{2}}\frac{{\left(j!\right)}^{2}}{\left(j-q\right)!{\left(q!\right)}^{2}}|x_{k}{|}^{2p}{\left|x_{k}\right|}^{2q}S^{p}S^{q}N^{\left(i-p\right)}N^{\left(j-q\right)}=\\ \;\;\;\;=\sum_{p=0}^{i}\sum_{q=0}^{j}\frac{{\left(i!\right)}^{2}}{\left(i-p\right)!{\left(p!\right)}^{2}}\frac{{\left(j!\right)}^{2}}{\left(j-q\right)!{\left(q!\right)}^{2}}\left(\frac{1}{K}\sum_{k=0}^{K-1}|x_{k}{|}^{2p}{\left|x_{k}\right|}^{2q}\right)S^{p}S^{q}N^{\left(i-p\right)}N^{\left(j-q\right)}. \end{array}$$

For $x_{k}=e^{j\phi_{k}}$, we have an exact expression valid even for small *K* (we do not need to assume K→∞), and we obtain

$$\begin{array}{cc} \frac{1}{K}\sum_{k=0}^{K-1}E\left[|y_{k}{|}^{2(i+j)}\right] & =\sum_{p=0}^{i+j}\frac{{\left(\left(i+j\right)!\right)}^{2}}{\left(i+j-p\right)!{\left(p!\right)}^{2}}S^{p}N^{\left(i+j-p\right)}=\mu_{2(i+j)}. \end{array}$$

and

(A10) $$\begin{array}{cc} \frac{1}{K}\sum_{k=0}^{K-1}E\left[|y_{k}{|}^{2i}\right]E\left[|y_{k}{|}^{2j}\right] & =\sum_{p=0}^{i}\sum_{q=0}^{j}\frac{{\left(i!\right)}^{2}}{\left(i-p\right)!{\left(p!\right)}^{2}}\frac{{\left(j!\right)}^{2}}{\left(j-q\right)!{\left(q!\right)}^{2}}S^{p}S^{q}N^{\left(i-p\right)}N^{\left(j-q\right)} \end{array}$$

(A11) $$\begin{array}{cc} \; & =\mu_{2i}\mu_{2j}. \end{array}$$

In summary, we have that, for signals in the form of complex exponential with deterministic phase sequence, the elements of the covariance matrix are given by

(A12) $${\left[C_{T}\right]}_{ij}=\frac{1}{K}\left(\mu_{2(i+j)}-\mu_{2i}\mu_{2j}\right).$$

Note that we obtain the same result as in the general random signal case and that Equation (A12) holds for all *K*. This is not true in all cases, as it represents a specific result due to the specific form of the deterministic signal $x_{k}$. In general, the elements of the covariance matrix in the case of deterministic signal is not given by Equation (A12) because clearly

(A13) $$\frac{1}{K}\sum_{k=0}^{K-1}|x_{k}{|}^{2p}{\left|x_{k}\right|}^{2q}\neq \left(\frac{1}{K}\sum_{k=0}^{K-1}{\left|x_{k}\right|}^{2p}\right)\left(\frac{1}{K}\sum_{k=0}^{K-1}{\left|x_{k}\right|}^{2q}\right).$$

It is still possible to write the covariance matrix in the general case, but its expression requires derivation of the terms

(A14) $$\frac{1}{K}\sum_{k=0}^{K-1}{\left|x_{k}\right|}^{2p}$$

and

(A15) $$\frac{1}{K}\sum_{k=0}^{K-1}|x_{k}{|}^{2p}{\left|x_{k}\right|}^{2q}$$

that depends obviously on signal $x_{k}$ and typically is only possible under the assumption that K→∞. For an example, see [10], where the covariance matrix for deterministic real sinusoids has been derived.

## Appendix B. Derivation of CRLBs for ${\hat{\mu }}_{2m}$

The CRLBs for ${\hat{\mu }}_{2m}$ can be derived from the expression of even-order moments given above through the derivatives of $\mu_{2m}$ with respect to *S* and *N*. We have for random signals

(A16) $$\begin{array}{cc} \frac{\partial }{\partial S}\mu_{2m}\left(S,N\right) & =\frac{\partial }{\partial S}\left(\sum_{n=0}^{m}\frac{{\left(m!\right)}^{2}}{\left(m-n\right)!{\left(n!\right)}^{2}}E[|x_{k}{|}^{2n}]S^{n}N^{\left(m-n\right)}\right) \end{array}$$

(A17) $$\begin{array}{cc} \; & =\sum_{n=0}^{m}\frac{{\left(m!\right)}^{2}}{\left(m-n\right)!{\left(n!\right)}^{2}}E[|x_{k}{|}^{2n}]\frac{\partial }{\partial S}\left(S^{n}\right)N^{\left(m-n\right)} \end{array}$$

(A18) $$\begin{array}{cc} \; & =\sum_{n=0}^{m}\frac{{\left(m!\right)}^{2}}{\left(m-n\right)!{\left(n!\right)}^{2}}E[|x_{k}{|}^{2n}]nS^{n-1}N^{\left(m-n\right)} \end{array}$$

(A19) $$\begin{array}{cc} \; & =\sum_{n=0}^{m}\frac{{\left(m!\right)}^{2}n}{\left(m-n\right)!{\left(n!\right)}^{2}}E[|x_{k}{|}^{2n}]S^{n-1}N^{\left(m-n\right)} \end{array}$$

Analogously, we have

(A20) $$\begin{array}{cc} \frac{\partial }{\partial N}\mu_{2m}\left(S,N\right) & =\frac{\partial }{\partial N}\left(\sum_{n=0}^{m}\frac{{\left(m!\right)}^{2}}{\left(m-n\right)!{\left(n!\right)}^{2}}E[|x_{k}{|}^{2n}]S^{n}N^{\left(m-n\right)}\right) \end{array}$$

(A21) $$\begin{array}{cc} \; & =\sum_{n=0}^{m}\frac{{\left(m!\right)}^{2}}{\left(m-n\right)!{\left(n!\right)}^{2}}E[|x_{k}{|}^{2n}]S^{n}\frac{\partial }{\partial N}\left(N^{\left(m-n\right)}\right) \end{array}$$

(A22) $$\begin{array}{cc} \; & =\sum_{n=0}^{m}\frac{{\left(m!\right)}^{2}}{\left(m-n\right)!{\left(n!\right)}^{2}}E[|x_{k}{|}^{2n}]S^{n}\left(m-n\right)N^{\left(m-n-1\right)} \end{array}$$

(A23) $$\begin{array}{cc} \; & =\sum_{n=0}^{m}\frac{{\left(m!\right)}^{2}\left(m-n\right)}{\left(m-n\right)!{\left(n!\right)}^{2}}E[|x_{k}{|}^{2n}]S^{n}N^{\left(m-n-1\right)} \end{array}$$

The CRLB is obtained as

(A24) $$\mathrm{var}\left({\hat{\mu }}_{2m}\right)\geq \left(\begin{array}{cc} \frac{\partial \mu_{2m}}{\partial S} & \frac{\partial \mu_{2m}}{\partial N} \end{array}\right)I^{-1}\left(S,N\right){\left(\begin{array}{cc} \frac{\partial \mu_{2m}}{\partial S} & \frac{\partial \mu_{2m}}{\partial N} \end{array}\right)}^{T}$$

that can be written as

(A25) $$\mathrm{var}\left({\hat{\mu }}_{2m}\right)\geq {\left(\frac{\partial \mu_{2m}}{\partial S}\right)}^{2}\frac{2SN}{K}+{\left(\frac{\partial \mu_{2m}}{\partial N}\right)}^{2}\frac{N^{2}}{K}.$$
